# Reducing Hispanic Children's Obesity Risk Factors in the First 1000 Days of Life: A Qualitative Analysis

**DOI:** 10.1155/2015/945918

**Published:** 2015-03-22

**Authors:** Jennifer A. Woo Baidal, Shaniece Criss, Roberta E. Goldman, Meghan Perkins, Courtney Cunningham, Elsie M. Taveras

**Affiliations:** ^1^Division of Gastroenterology and Nutrition, Boston Children's Hospital, 300 Longwood Avenue, Boston, MA 02115, USA; ^2^Division of General Academic Pediatrics, Department of Pediatrics, Massachusetts General Hospital, 100 Cambridge Street, 15th floor, Boston, MA 02114, USA; ^3^Department of Social and Behavioral Sciences, Harvard School of Public Health, 677 Huntington Avenue, Kresge Building, Boston, MA 02115, USA; ^4^Warren Alpert Medical School, Brown University, 111 Brewster Street, Pawtucket, RI 02860, USA

## Abstract

*Objectives*. Modifiable behaviors during the first 1000 days (conception age 24 months) mediate Hispanic children's obesity disparities. We aimed to examine underlying reasons for early life obesity risk factors and identify potential early life intervention strategies. *Methods*. We conducted 7 focus groups with 49 Hispanic women who were pregnant or had children < age 24 months. Domains included influences on childhood obesity risk factors and future intervention ideas. We analyzed data with immersion-crystallization methods until no new themes emerged. *Results*. Themes included coping with pregnancy may trump healthy eating and physical activity; early life weight gain is unrelated to later life obesity; fear of infant hunger drives bottle and early solids introduction; beliefs about infant taste promote early solids and sugary beverage introduction; and belief that screen time promotes infant development. Mothers identified physicians, nutritionists, and relatives as important health information sources and expressed interest in mobile technology and group or home visits for interventions. *Conclusion*. Opportunities exist in the first 1000 days to improve Hispanic mothers' understanding of the role of early life weight gain in childhood obesity and other obesity risk factors. Interventions that link health care and public health systems and include extended family may prevent obesity among Hispanic children.

## 1. Introduction 

Emerging national data suggests a plateau in obesity prevalence among children in the USA [1]. Yet all age groups continue to have high obesity prevalence, with 16.9% US children aged 2–12 years affected by obesity [1]. Socioeconomic and racial/ethnic disparities in childhood obesity persist and appear to be widening [1–4]. Hispanic children of age 2–5 years have a fivefold higher prevalence of obesity compared to non-Hispanic white counterparts, suggesting that the etiologies of disparities in childhood obesity start early in life [5, 6].

The first 1000 days of life—conception through 24 months of age—are recognized as a critical period for optimal nutrition and development [7]. Racial/ethnic differences in modifiable risk factors for childhood obesity exist during pregnancy, infancy, and early childhood [8–12]. Among Hispanic children, nonexclusive breastfeeding, early introduction of solid foods, sugar-sweetened beverage intake, screen time, insufficient sleep, and other modifiable behaviors during infancy and early childhood contribute substantially to racial/ethnic disparities in mid-childhood obesity [13]. The underlying reasons for these racial/ethnic differences in early life risk factors are unknown. In order to reduce racial/ethnic disparities in childhood obesity, root origins of obesity risk factors and unique approaches to prevent obesity in Hispanic children during the first 1000 days of life must be identified.

The overall goals of this qualitative study were to examine the underlying reasons for early life obesity risk factors during pregnancy, infancy, and early childhood among Hispanic families and to identify intervention strategies that have the potential to reduce obesity risk factors early in life. Qualitative methods are uniquely suited to reveal beliefs that lead to different behaviors in minority populations, and focus group discussions allow for observation of psychosocial dynamics that would not be identified in interviews. We aimed to identify beliefs and perceptions of pregnancy health; perceptions of and influences on infant and child weight gain, diet, screen time, and insufficient sleep; maternal explanatory factors for childhood obesity; and potentially effective future obesity prevention intervention approaches among Hispanic families.

## 2. Methods

### 2.1. Study Setting and Participants

We conducted a total of 7 semistructured focus group discussions with Hispanic mothers at three life stages: 2 groups of women during pregnancy, 3 groups of mothers of infants (birth to age <7 months), and 2 groups of mothers of children in early childhood (age 7 months to 24 months). Women with a prenatal visit for a singleton gestation were eligible for pregnancy groups. Parents (mothers or fathers) of children between birth and age 6.9 months were eligible for enrollment in infancy groups, and those with children of age 7 through 24 months were eligible for early childhood groups. Pregnant women and parents of infants and children under 2 years of age who could respond to questions in English or Spanish were eligible for recruitment. We excluded families for whom (1) the pregnant woman or eligible caretaker was under 18 years of age, (2) the pregnant woman or child had chronic medical conditions that interfered with growth, nutrition, or physical activity, or (3) the health care provider thought study participation was inappropriate.

Research staff recruited patients who had an outpatient visit for routine prenatal or pediatric care at a federally qualified community health center (CHC) with a multispecialty provider group in eastern Massachusetts that serves a racial/ethnically and socioeconomically diverse population. A total of 96 eligible parents were recruited for focus groups, among which 18 were ineligible, 29 declined to participate (16 uninterested, 8 unavailable at time of focus group, 3 unable to find child care, and 2 unable to obtain transportation), and 49 elected to participate. We provided $40 for participation and $20 to reimburse for childcare and travel.

### 2.2. Data Collection

The research team developed a focus group discussion guide over multiple meetings.  Table 1 shows discussion guide domains which included (1) perceptions of weight gain during respective life stage; (2) explanatory factors for development of childhood obesity; (3) beliefs about obesity risk factors; and (4) perceptions of potential future interventions.

We conducted all focus groups at the CHC between July 2013 and January 2014. We set an initial target of 6 focus group discussions (2 for each life course stage), but we added a third infancy group for consistency in sample size across life course stages. At recruitment, each participant completed a brief survey asking general demographic questions such as age, educational attainment, and number of children in household. For each focus group, a professional bilingual moderator, whose race/ethnicity was concordant with the participants', facilitated the discussions to enhance the comfort level of participants. The same moderator conducted all focus groups. The moderator was trained on study aims and discussion guide questions through a half-day session and recurrent study team phone calls. The focus groups were primarily in Spanish with some English interpretation. Bilingual study staff members took notes during discussions, and team members debriefed after each focus group. Discussions were audio-recorded and transcribed verbatim in Spanish, and then professionally translated to English. Participants were assigned random numbers to replace identifying information and protect confidentiality. The institutional review board at Massachusetts General Hospital* for* Children approved the study protocols. All participants provided informed, written consent prior to participation.

### 2.3. Analytic Approach

We conducted content analysis of the transcribed focus group discussions using immersion-crystallization techniques [14]. Immersion-crystallization is an iterative analytic approach with two stages: immersion and crystallization. During the immersion process, researchers immerse themselves in the data collected by reading data in detail. During crystallization, researchers analyze the data examined during the immersion process for important patterns and themes. The immersion and crystallization processes are continued until no new patterns or themes emerge. Specifically, research team members (JWB, SC, CC, and REG) read the transcripts and discussed the data as a group repeatedly to determine topical content and emerging themes. Research team members took detailed notes during meetings. Following the immersion-crystallization processes, we developed and refined a codebook through iterative discussions. We used NVivo 10 software to import transcripts, code the data, and organize codes. Two members of the research team (SC and CC) coded one transcript and reviewed consistency of coding to ensure consensus on categorization of the data. One member of the team (SC) coded all remaining transcripts. We then used the code reports to continue content analysis and interpretation of themes [15]. We continued analysis until no new major themes emerged and we resolved discrepancies at research team meetings. The bilingual focus group moderator and study staff present at focus group discussions agreed with data interpretation.

## 3. Results

### 3.1. Participant Characteristics

Table 2 shows characteristics of the 49 mothers who participated. Mean maternal age was 26.4 (SD 6.6) years. Mean gestational age in pregnancy groups was 5.1 (SD 1.8) months. Mean child age was 2.8 (SD 2.0) months in infancy groups and 14.3 (5.3) months in early childhood groups. More than half of the women were born outside USA, and most spoke both Spanish and English.

### 3.2. Beliefs and Perceptions of Pregnancy Health

Table 3 shows themes related to pregnancy health.

*Mothers Coping with Their Own Physical Changes during Pregnancy May Trump Healthy Eating and Physical Activity.* Most women in all seven groups believed that the keys to a healthy pregnancy lie in healthy eating and physical activity. Many women in the pregnancy groups reported improving dietary habits during pregnancy by consuming a balanced diet of fruit, vegetables, dairy, and lean proteins, while avoiding fast food and soda. However, some reported that nausea made tolerating healthy foods difficult, saying “Most food hurts me, so I feel like my stomach is heavy…vegetables, that I know are good and I've always liked, they make me feel like vomiting” (Pregnancy Group). Instead cravings or nausea led to consumption of foods that they perceived as unhealthy (i.e., fried and salty foods and candy).

In all seven groups, most women noted that physical activity during pregnancy is important to improve comfort, reduce duration of labor, and enhance maternal energy and health. Many women reported lack of physical activity because they were tired, more easily fatigued, or felt uncomfortable with their pregnancy weight. One woman said, “I am not doing any physical activity because of laziness. Because before I was studying. I went back and forth on foot. And now that I'm done studying—and I say I'm not going to get out of bed” (Pregnancy Group). Although some women believed that physical activity during pregnancy could improve offspring's health, none linked it to prevention of excessive gestational weight gain or childhood obesity.

*Weight Extremes Should Be Avoided during Pregnancy.* Most women believed that it is possible to have excessive gestational weight gain, and some equated its health risks to those of insufficient weight gain. Perceived complications of excessive gestational weight gain included difficult labor, need for cesarean section, and maternal health complications. None identified maternal weight status or gestational weight gain as a risk factor for childhood obesity. All women had heard the popular expression that “pregnant women are eating for two” and none agreed with it. Several thought it was important to eat a variety of healthy foods to provide the right nutrients to their baby, rather than to gain enough weight for two people. One woman stated, “It's not eating for two, but knowing how to eat, for the baby you're carrying” (Pregnancy Group).

### 3.3. Perceptions of and Influences on Infant and Child Weight Gain, Feeding, Screen Time, and Sleep

Table 3 includes themes related to infant and child weight gain, feeding, screen time, and sleep.

*Excess Child Weight Gain in First Years of Life Is Possible but Inconsequential.* In infancy and early childhood groups, almost all mothers believed that infants and children under age 2 years could gain too much weight. When asked, several mothers disagreed with the popular saying “a chubby baby is a healthy baby.” A few mothers in the infancy and early childhood groups did worry about their infant or child's risk for obesity and discussed their desire to prevent their infant from becoming obese. However, most mothers believed that excess weight in this age group, while possible, was not a problem unless specifically told otherwise by their pediatrician, stating that “you feed them what they want, and later when they start getting older they'll start knocking off some of the pounds” (Infancy Group).

Many mothers in infancy and early childhood groups had discussed their child's weight with a pediatrician and reviewed their child's growth charts. Most mothers reported that pediatricians are a trusted source of information on child weight gain. However, a few mothers had been told that their child was obese and did not believe the pediatrician. They either cited their own instincts as a mother as better than the doctor's medical knowledge or their own belief that there are not consequences to excess weight early in childhood.

*Maternal Fear of Infant Hunger Drives Nonexclusive Breastfeeding and Addition of Solid Foods to Bottles.* Although mothers in pregnancy, infancy, and early childhood groups identified exclusive breastfeeding as the best option for feeding their newborn, some mothers in infancy groups feared that breastfeeding did not provide sufficient nutrition for their baby. Several women reported concerns that inadequate breast milk production left their infant hungry, and a few women pumped to check the volume of breast milk produced. According to one mother, “I feel like I'm not giving her enough…because I do not see the amount coming out…when you do formula, you know how much you're giving…so I started pumping to see how much I could pump” (Infancy Group). Fear regarding inadequate breast milk volume led to supplemental or exclusive bottle feedings by some mothers.

Despite most mothers recognizing that solid foods should be introduced at or around 4–6 months of age, solids in the form of cereal, purees, or sugar added to bottles appeared to be an exception to this rule. “I've given my son, at three months old, rice cereal in his formula. There's nothing wrong with it in my opinion because the rice cereal has iron, it has protein, it has vitamins…And he sleeps more at night,” according to one mother (Early Childhood Group). Mothers also reasoned that adding solids to bottles helped infants feel full, gain more weight, calm down, and receive adequate nutrition. Female family members were common sources of advice and example on feeding routines, including adding cereal and other solid foods to bottles.

*Mothers Feel Responsible for Ensuring Infant Satiety.* All mothers in all infancy and early childhood groups believed that they were attuned to their child's hunger and satiety cues. Several mothers believed that parents must feed their child under age 6 months until they were full. One mother explained, “I think that if he's younger than six months you have to [feed] him until you think [he's full] since they do not know, but from six months on, they—they know how much they want, and they make [it] known” (Early Childhood Group). Most mothers in infancy groups believed it was important for children to be full and cited satiety cues of losing interest in feeding, having a hard stomach, pushing the bottle away with hand or tongue, sighing, or falling asleep. Mothers in infancy groups commonly noted infant hunger cues of sucking/rooting, putting hand in mouth, and crying. In infancy groups, all mothers thought on-demand feeding was preferable. However, several still kept track of time between feeds, woke infants to feed, or fed infants while they were asleep.

Although most mothers in the early childhood groups showed confidence in their child's ability to express a desire for certain food or drink compared to when they were infants, they also noted that children might ask for food or drink for reasons other than hunger. “Sometimes kids want to keep eating and actually they do not need it…we mothers have to be very careful…because the baby can…gain weight that is not appropriate…that's what I always ask when I go to the nutritionist, is she OK for her age?” (Early Childhood Group). Mothers worried that eating in the absence of hunger could lead to childhood obesity, and they received advice from the Special Supplemental Nutrition Program for Women, Infants, and Children (WIC) nutritionists on portion sizes and balanced meals.

*Family Beliefs about Infant Taste Development Promote Early Introduction of Solid Foods and Sugar-Sweetened Beverage Intake.* Almost all mothers in infancy and early childhood groups described family beliefs that infants should experience a variety of foods to develop taste preferences, some starting as young as 1 month of age. Most reported that her child's grandmothers, aunts, fathers, and other male relatives “want to give her beans, rice, they want to give her food, on the contrary, not milk, food” (Infancy Group). Relatives fed young infants chocolate, fruits, lollipops, cookies, meats ground with beans or rice, and sports beverages. A few mothers gave their young children sports drinks or sodas because they perceived that after 1 year of age it is important to teach children to “taste.” One mother explained, “I give her juice, and she's also tried soda. They have to try everything…so she (learns) the taste of everything” (Early Childhood Group). While a few mothers and reportedly many extended family members believed “tastes” of these foods would teach the child to eat or not eat certain foods, others, mainly male relatives, reportedly had a desire to provide larger food portions to make the child stronger. Most mothers expressed frustration with these practices, and several worried they could lead to development of illness, such as diarrhea. Most mothers did not feel empowered to change their relative's behaviors, but many did try to discuss their concerns with family members.

*Variation in Maternal Knowledge about Healthy Beverage Choices for Children.* Mothers stressed a preference for providing homemade or 100% juice as “natural” beverage options for children. “Well if it's 100 percent they are [healthy]… I make him his juice from the fruit itself, natural” (Early Childhood Group). Several reported diluting juice with water based on advice from pediatricians or WIC providers. Many mothers did not give their children soda, and a few participants discussed reading labels to avoid giving children beverages with added sugar. However, several mothers in early childhood groups did not distinguish between fruit drinks, sports drinks, and punch. Notably, one mother perceived that, unlike juice, sports drinks did not make her child hyperactive, stating “I give him more [sports drinks] than juice…because [sports drinks] contains minerals that the body needs…, and he does not get active or very hyperactive like [with] juices.” Some mothers worried that giving juice or sugar-sweetened beverages to their child could make them hyperactive, but none spoke about concerns for sugar-containing beverages leading to obesity or other adverse health outcomes.

*Maternal Belief That Screen Time in the First Two Years of Life Is Important for Child Development.* All mothers in all infant and early childhood groups disagreed with the recommendation that children of age 2 years should not watch television, and some felt very strongly that television viewing was important for their child's visual and cognitive development. One mother stated, “It's good because she watches [learning videos] and then she learns more” (Infancy Group).

All mothers reported that their infants and young children watched television regularly, most for 1-2 hours daily. Some mothers reported that their infant or child routinely went to sleep while watching television, explaining “I have to put on the TV for her to fall asleep because she started watching television at three months and I would take her out and she cried, cried, cried” (Infancy Group). No mother reported being told by their pediatrician that their child should not watch television. Even if a pediatrician were to advise that their infant or child should not watch television, several mothers said they would seek additional information from other sources or would want evidence of a negative impact on child vision or cognitive development before they would reduce or eliminate their child's screen time. No mothers identified screen time as a risk factor for childhood obesity.

*Sleep Routines Should Start in Early Life.* In the infancy groups, mothers reported their children slept 10–16 hours, but all agreed that infants should sleep 14–16 hours over a 24-hour period. Most mothers in infancy groups thought sleep routines should start between 2–4 months of age, but several thought they should start after 6 months of age. In early childhood groups, mothers thought sleep routines should start at birth. Family members, the Internet, and television shows were cited by a few as sources of advice. “My grandfather would tell me that ever since he was born I should get him used to sleeping at night,” according to one mother (Infancy). No one mentioned discussing child sleep routines with their pediatrician. In order to get their children to fall asleep, mothers changed positions, rocked them, gave them baths, snuggled with them, or gave them food or milk because they believed a full stomach would allow longer sleep. A few mothers thought that daytime physical activity was key to their child getting a good night's sleep. No mother recognized that curtailed sleep is a risk factor for childhood obesity.

### 3.4. Maternal Explanatory Factors for Childhood Obesity

Table 3 shows themes related to maternal explanatory factors for childhood obesity.

*Maternal Belief That Early Life Weight Gain Impacts Health but Is Unrelated to Later Life Obesity.* Many women in pregnancy groups believed that excessive gestational weight gain has negative impacts on infant health but did not include childhood obesity as a potential outcome. Many women discussed believing that maternal weight status and pregnancy weight gain do not contribute to childhood obesity and that child nutrition after birth was the most important contributor to childhood obesity. In infancy groups and early childhood groups, most mothers believed children could gain too much weight during the first 2 years of life.

In each life course period, most mothers did not identify early life weight gain as a risk factor for childhood obesity later in life. Mothers expressed a sense of hopelessness in being able to control future child weight status, and one said “I think the appetite could change… I'm assuming that it just depends on how you grow yourself… I think it's the baby. Not exactly what you're doing” (Infancy Group). Some women also demonstrated a lack of awareness that obesity could herald future diabetes risk, and a few women thought that diabetes could lead to obesity. One mother stated, “You cannot really control [development of childhood obesity]…if you have diabetes when you're pregnant, all kids are bound to be big, and then you have some [pregnant women] that eat a lot, and the babies come out five pounds, so it's like you cannot really control it” (Pregnancy Group). Several women strongly believed that the only explanation for overweight or obesity in young children would be a medical cause.

*Overfeeding and Early Introduction of “Adult” Foods Lead to Childhood Obesity.* In infancy and early childhood groups, mothers most commonly discussed feeding practices as the etiology of childhood obesity. Some mothers explained that overfeeding early in life could lead to a larger appetite or even an irreversible and potentially dangerous expansion of children's stomachs stating that “I know people who have damaged their stomachs giving them food, feeding them… giving them food that is not [right] for their bodies and they open the belly with, it gives them more extra room” (Infancy). Mothers cited an unhealthy diet as another cause for childhood obesity.

Several mothers also noted that family and cultural influences on introduction of table foods before 6 months of age could lead to excess weight gain. “Dominicans—I include myself—for excess weight—that they feed him too much at a very early age, so they encourage him to eat from the time [they're] small. Food—rice, beans, meat…way before [6 months of age]” (Early Childhood). However, many mothers disagreed with this idea and did not believe that early introduction of solid foods caused obesity.

### 3.5. Potential Effective Childhood Obesity Prevention Interventions Strategies

Mothers identified topics and strategies for future interventions to prevent childhood obesity among Hispanic families. Mothers were interested in learning more about feeding and nutrition (including breastfeeding, formula selection, and how to prepare baby food at home), sleep routines and sleep training, child weight gain, and normal child development. For topics related to screen time, mothers felt strongly that they needed more information that demonstrated a negative impact on vision or cognitive development in order to make behavior changes.

Almost all mothers showed interest in group classes to learn more about caring for their child. Mothers valued not just information but also hearing the experience of other mothers. Most mothers also wanted more easily accessible information from pediatricians. Text messaging, telephone support, email, health coaches, WIC parenting groups, and established Internet sources were reported as potential avenues for interventions. A few mothers were interested in home visits, and several reported interest in mailed brochures to reduce reliance on Internet access and enhance the accessibility of information for family members.

Family members were viewed as stubborn and difficult to change. Because of the influence of relatives on child feeding practices and childcare, some mothers noted that interventions should not just target mothers, they should also include fathers and other family members. Some mothers thought that interventions that provided evidence-based information in print or on the Internet would be the most convincing way to change their family members' habits.

## 4. Discussion 

In this qualitative study of Hispanic mothers with children in the first 1000 days of life, we found that mothers were unaware of the critical role that weight status from gestation through 2 years of age plays in future development of childhood obesity. Mothers did not recognize that early introduction of cereals and purees, screen time, or insufficient sleep are modifiable early life risk factors for childhood obesity. We found that mothers desired childhood obesity prevention interventions based on health care and public health systems and wanted interventions to include extended family members.

Our study provides information about the origins of risk factors for childhood obesity and highlights opportunities to intervene during the earliest moments of life to prevent development of obesity risk factors among Hispanic families, a population that carries disproportionate burden of childhood obesity. Black non-Hispanic children are also affected by disparities in childhood obesity, and a recent qualitative study by Herring et al. similarly found that pregnant African-American women believed that physical symptoms of pregnancy impact gestational diet quality and high gestational weight gain is bad for maternal health [16]. However, unlike our findings, African-American women in Herring's study had many perceptions that encouraged high gestational weight gain and they did not believe that high gestational weight gain is harmful for infants [16]. Our study provides information to inform future culturally appropriate health messages for Hispanic women during pregnancy and early life.

Similar to our study, a prior content analysis of motivational counseling phone calls among predominantly non-Hispanic white women during the postpartum period found that mothers identified crying and fussiness as signs of hunger, supplemented with bottles immediately after feeds to ensure fullness, and fed their children to soothe overnight [17]. Our results suggest these feeding practices, which can promote overfeeding, may cross cultural boundaries.

Our research also extends previous findings among older Hispanic children to earlier stages in the life course. In a recent qualitative study of Mexican origin mothers with elementary school-aged children, Martinez et al. found that mothers felt primarily responsible for ensuring their children were well-fed [18]. In our study, we identified that other family members, such as fathers and grandmothers, are influential in child feeding patterns that increase childhood obesity risk. Similar to our results, another qualitative study in 2005-2006 by Lindsay et al. found that family members, particularly grandmothers, challenged eating habits that Hispanic mothers set for their children [19]. We also learned that mothers had limited confidence in their ability to influence behavioral change among other family members.

Unlike Lindsay et al., who found that Hispanic mothers believed that children should weigh more [19], we found that Hispanic mothers largely did not endorse the popular notion that “a chubby baby is a healthy baby.” Social desirability bias could explain our findings, though it is more likely that our findings represent a shift in weight perception norms over the past several years secondary to widespread publicity on the dangers of obesity for adults and children.

Two of our findings were particularly striking. First, incorrect family beliefs surrounding infant taste development and satiety, as well as concerns regarding inadequate nutritional quality of breast milk and infant formula, promoted early introduction of solid foods. Introducing solid foods earlier than 4 months of age is an established risk factor for childhood obesity [12, 20] and accounts in part for Hispanic disparities in childhood obesity [8, 21]. Second, we found that all mothers firmly felt that screen time was important to learning and visual development for their infants and young children. Families also used screens to put their infants to sleep. A recent qualitative study on television use among Boston-area Hispanic and black non-Hispanic families with young children also found that families were unaware of the adverse health outcomes associated with television use [22]. Child screen time is associated with obesity, sleep disturbance, attention issues, and language delays [23]. The American Academy of Pediatrics discourages media use by children under age 2 years and recommends that pediatricians discuss these recommendations with parents [23]. Future obesity prevention interventions with Hispanic families should include culturallytailored messages to correct these beliefs and reduce early introduction of solids foods and screen time among infants and young children, including health communication insights.

Most existing qualitative research surrounding childhood obesity in Hispanic families has focused on feeding practices in school-aged children. Our study is unique in its inclusion of families at different stages in the life course and its examination of etiologies of established early life risk factors for obesity that are both dietary and nondietary in nature. Our study does have limitations. The participant sample is geographically restricted and thus could limit the ability to generalize our results. Also, all focus group participants were mothers and our findings do not include perspectives of fathers. However, any parent was eligible for enrollment and the ultimate inclusion of only mothers was likely a result of their role as primary caretakers in these families.

In summary, opportunities exist in the first 1000 days of life to improve Hispanic mothers' understanding of childhood obesity risk factors and the role of early life weight gain in later life obesity. Mothers identified interventions that link health care systems with public health systems, use multiple methods of delivery for intervention components, and include extended family members as potentially effective approaches to reduce obesity risk factors for Hispanic children early in life. Future interventions that prevent the development of obesity risk factors in the earliest stages of life and consider the social-contextual environment may reduce racial/ethnic disparities in childhood obesity and its adverse outcomes.

## Figures and Tables

**Table 1:** Focus group discussion guide domains according to focus group type.

Domain	Pregnancy groups	Infancy groups	Early childhood groups
Health information sources	✓	✓	✓
Beliefs about weight gain during respective life course period	✓	✓	✓
Beliefs about and influences on formation of obesity risk factors			
(i) Infant/child bottle use and breastfeeding	✓	✓	✓
(ii) Introduction of solid foods to infant/child		✓	✓
(iii) Infant/child ability to self-regulate feeding		✓	✓
(iv) Infant/child sleep		✓	✓
(v) Infant/child screen time		✓	✓
(vi) Sugar-sweetened beverage intake and diet quality during respective life course period	✓	✓	✓
(vii) Physical activity during pregnancy	✓		
Parental explanatory factors for childhood obesity	✓	✓	✓
Prospective future interventions to reduce childhood obesity	✓	✓	✓

**Table 2:** Mother and child characteristics according to focus group discussion participation. Data from 49 Hispanic mothers.

	Pregnancy groups (*N* = 17)	Infancy groups (*N* = 15)	Early childhood groups (*N* = 17)
Parent/family characteristic			
Maternal age, years, and mean (SD)	25.6 (6.4)	25.6 (7.5)	27.9 (6.1)
Education, high school graduate, *n* (%)	13 (76%)	9 (60%)	12 (71%)
Nulliparous, *n* (%)	14 (82%)	10 (67%)	6 (35%)
Married/cohabiting, *n* (%)	11 (65%)	9 (60%)	10 (59%)
US-born, *n* (%)	6 (35%)	7 (47%)	6 (35%)
Language comfort			
Spanish-only	9 (53%)	4 (27%)	3 (18%)
Either English or Spanish	8 (47%)	11 (73%)	13 (76%)
Gestational age (months)	5.1 (1.8)	n/a	n/a
Child characteristics			
Age (months)	n/a	2.8 (2.0)	14.3 (5.3)

**Table 3:** Themes related to weight gain and obesity risk factors in pregnancy, infancy, and early childhood among Hispanic women. (*n* = 49).

Beliefs and perceptions of pregnancy health	
(i) Coping with own physical changes during pregnancy may trump healthy eating and physical activity (ii) Weight extremes should be avoided during pregnancy	

Perceptions of and influences on infant and child weight gain, feeding, screen time, and sleep	
(i) Excess child weight gain in first years of life is possible but inconsequential (ii) Maternal fear of infant hunger drives nonexclusive breastfeeding and addition of solid foods to bottles (iii) Mothers feel responsible for ensuring infant satiety (iv) Family beliefs about infant taste promote early introduction of solid foods and sugar-sweetened beverage intake (v) Variation in maternal knowledge about healthy beverage choices for children (vi) Maternal belief that screen time in the first two years of life is important for child development (vii) Sleep routines should start in early life	

Maternal explanatory factors for childhood obesity	
(i) Maternal belief that early life weight gain impacts health but is unrelated to later life obesity (ii) Overfeeding and early introduction of “adult” foods lead to childhood obesity	
